# The need for improved Australian data on social determinants of health inequities

**DOI:** 10.5694/mja2.51695

**Published:** 2022-08-25

**Authors:** Joanne Flavel, Connie Musolino, Toby Freeman

**Affiliations:** ^1^ Stretton Health Equity University of Adelaide Adelaide SA; ^2^ Flinders University Adelaide SA

**Keywords:** Data collection, Population characteristics, Social determinants of health

## Open access

Open access publishing facilitated by The University of Adelaide, as part of the Wiley ‐ The University of Adelaide agreement via the Council of Australian University Librarians.

## Competing interests

No relevant disclosures.

in reply:

We thank Fortune and colleagues^1^ for highlighting the shortcomings of current Australian data for identifying inequities in social determinants and health outcomes for people with disability in response to our recently published article.^2^ We support their call for development of a nationally agreed, consistent disability identifier to build a strong evidence base for reducing health inequities.^1^

Robust data on experiences of people with disability and their health outcomes are needed to inform and evaluate policy. The collection and public reporting of such data are obligations under the United Nations Convention on the Rights of Persons with Disabilities.^3^ Currently, there are differences in definitions of disability across jurisdictions and data sources.^4^ These differences limit data comparability when presenting data from different sources.^5^ There are also data gaps, including no national data source for some health and wellbeing indicators for people with disability, data sources that include a disability identifier but do not include some groups of people with disability (particularly in very remote areas or non‐private dwellings), under‐representation of people with disability who cannot answer survey questions without support, and absence of disability indicators in some data sources.^5^ A consistent national disability indicator that enables disaggregation by disability would overcome some but not all these data gaps.

We agree with Fortune et al that the National Disability Data Asset provides an opportunity to monitor inequities in health and social determinants of health for people with disability. Advances in data linkage, coupled with a consistent national disability identifier would improve understanding of the experiences of people with disability. In addition, efforts should be made to collect data that are representative of all groups of people with disability for the full range of health and wellbeing indicators. With representative data, the identifier would allow examination of intersectionality between disability, socio‐economic status, and gender.^6^

We echo the call by Fortune and colleagues for meaningful involvement of people with disability, representative organisations and disability advocates in decision making concerning the development of a nationally consistent disability identifier, but also in the collection of data that bridge all current data gaps for people with disability.
